# Discerning protein pools by selective staining with self‐labeling tags

**DOI:** 10.1002/1873-3468.70425

**Published:** 2026-08-07

**Authors:** Kati Fischermanns, Johannes Broichhagen

**Affiliations:** ^1^ Leibniz‐Forschungsinstitut für Molekulare Pharmakologie (FMP) Berlin Germany

**Keywords:** fluorescence, imaging, multiplexing, protein labeling, receptor pools

## Abstract

Transmembrane‐spanning cell surface proteins undergo maturation from the site of synthesis in the endoplasmic reticulum to the plasma membrane, and may be endocytosed back into the cell. Microscopy allows the visual separation of such populations at the cell surface and in intracellular compartments and to monitor trafficking and dissect the relative abundance of such pools. Protein tags, like SNAP‐tag, HTP, and FAST, combined with their respective (im)permeable ligands fused to fluorescent dyes, open up new possibilities to distinguish between intra‐ and extracellular protein pools. Introducing cleavable groups in dye‐ligand molecules allows interrogation of endocytosed pools by stripping the remaining surface pools. Latest developments, for instance, fluorescence lifetime imaging microscopy and brightness demixing, give further opportunities in microscopy.

## Graphical abstract

Cell surface proteins have an intra‐ and extracellular pool. Combining genetic fusion to self‐labeling tags that can be addressed with small molecule fluorophores allows separating these pools. We highlight recent developments and techniques for state‐of‐the‐art interrogation of cell surface proteins in the complex tissue setting. Micrograph adapted from Roßmann et al. 2026 (https://doi.org/10.1038/s41467‐025‐68134‐0), under the terms of a Creative Commons CC BY 4.0 license.

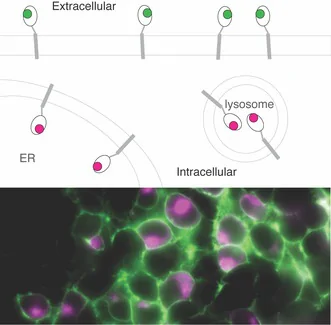

## Abbreviations

AF, AlexaFluor

AMPAR, α‐amino‐3‐hydroxy‐5‐methyl‐4‐isoxazolepropionic acid receptor

BG, *O* ^6^‐Benzylguanine

CA, chloroalkane

CLUSTER, chemical label‐unfold‐splice technology enables recombination

D2R, dopamine receptor D2

DNA‐PAINT, DNA‐points accumulation for imaging in nanoscale topography

dSTORM, direct stochastic optical reconstruction microscopy

ER, endoplasmic reticulum

F2X, difluorinated xanthene

FAST, fluorescence‐activating and absorption‐shifting tag

FLEXTAG, fluorescent labeling for exchangeable, X‐resilient tagging in advanced generic nanoscopy

FLIM, fluorescence lifetime imaging microscopy

FRET, Förster resonance energy transfer

GLP1(R), glucagon‐like peptide 1 (receptor)

GPCR, G protein‐coupled receptor

HBR, hydroxybenzylidene rhodanine

HTL, HaloTag ligand

HTP, HaloTag protein

JF, JaneliaFluor

MESNA, sodium 2‐mercaptoethane sulfonate

mGluR, metabotropic glutamate receptor

OG, Oregon Green

POI, protein of interest

SiR, silicon rhodamine

SS, disulfide bridge

STED, stimulated emission depletion

STORM, stochastic optical reconstruction microscopy

TM, transmembrane

TMR, tetramethylrhodamine

TrkB, tropomyosin receptor kinase B

## Protein pools and physiological implications of turnover

The human genome encodes for > 20 000 proteins, of which variants exist through splicing and post‐translational modifications, that serve a plethora of functions [1]. The expression pattern of proteins in each cell shapes its specific biological function and can be used to identify a certain cell type. But even among the same cell type, heterogeneity is created as expression patterns can vary [2, 3]. Even more, different protein pools emerge and shuffle due to a highly dynamic system [4]. While techniques such as scRNAseq or western blotting only give a snapshot of a protein potentially being transcribed or being expressed, respectively, microscopy is the method of choice to determine localization—nowadays down to the two‐digit nanometer level [5]. The aim of this review is to highlight recent advances in tagging strategies, chemical dye and ligand engineering, together with the developments in multiplexed imaging that paved the way for simultaneous investigation of several protein populations with subcellular precision.

Cell surface receptors that reach the extracellular space follow a highly regulated journey from the site of synthesis to the site of action, which is crucial for the proper functioning of a cell [6]. Proteins are synthesized in the endoplasmic reticulum (ER) where they undergo post‐translational modifications, such as *N*‐linked and *O*‐linked glycosylation, phosphorylation, and ubiquitination. These modifications enhance the stability and are crucial for trafficking and their functionality. Once functional conformation is achieved, they are packaged into vesicles and transported to the Golgi apparatus, where they undergo further processing. The last step of the secretory pathway involves enclosing them into transport vesicles for their delivery to the plasmalemma. Once receptors, mostly ion channels, receptor‐linked enzymes, and G protein‐coupled receptors (GPCRs), are integrated into the cell membrane, they are able to initiate signaling to sense outside stimuli for transmitting them to intracellular information (although signaling is not entirely restricted to the cell surface) [7, 8]. Upon activation and the initiation of signaling, internalization into endosomes usually follows, and the receptors are either targeted for lysosomal degradation or sorted for recycling to the surface. These processes create at least two distinct functional and non‐functional protein pools within the cell:

- The intracellular pool: Immature proteins residing in the ER and Golgi as well as mature proteins that have been internalized from the plasmalemma, targeted for lysosomal degradation or recycling.
- The extracellular exposed/cell surface pool: Fully processed proteins anchored in the plasmalemma, accessible to extracellular ligands and—in the case of receptors—ready to initiate signaling.

The importance of understanding the cellular and molecular principles underlying membrane trafficking pathways is an active field of research to combat diseases, such as neurological and metabolic disorders like Alzheimer's disease [9] and type 2 diabetes mellitus [10]. Although the cell types associated, neurons and pancreatic beta cells, are apart in the body and are morphologically different, they are both excitable and share a similar exocytotic machinery. Even more, type 2 diabetes has been shown to be connected to Alzheimer's disease as the resistance to insulin is a recognized risk factor for dementia [11].

Some cell surface receptors can be found in both cell types, exerting their functions. For instance, the subunits 2 and 3 of ionotropic receptor α‐amino‐3‐hydroxy‐5‐methyl‐4‐isoxazolepropionic acid receptor (AMPAR), an ion channel, the tropomyosin receptor kinase B (TrkB), a receptor‐linked enzyme, and the glucagon‐like peptide‐1 receptor (GLP1R), a GPCR, are commonly expressed, all of which undergo dynamic trafficking and have an impact on disease development [12, 13, 14, 15]. AMPARs undergo rapid constitutive and activity‐dependent trafficking and the strength of excitatory synaptic transmission is directly proportional to the density of AMPARs at the postsynaptic membrane [16] and their “turnover rate” is a key determinant of synaptic homeostasis [17]. In beta cells they are involved in insulin secretion by docking insulin granules [18]. To a similar extent, TrkB shapes memory and emotion via the regulation of synaptic plasticity. Disruptions of TrkB signaling or trafficking contribute to memory deficits and psychiatric disorders, rendering it a promising target for future therapeutics [14]. Deletion of TrkB from beta cells leads to impaired glucose tolerance and reduced insulin secretion [19]. The importance of the spatial separation of receptor pools is moreover highlighted by studies regarding the GLP1R. The receptor is widely known as target for the treatment of type 2 diabetes and obesity as it regulates the release of insulin in pancreatic beta cells under high glucose conditions, and regulates satiety within the hypothalamus [20]. Once activated by an agonist, for example endogenous glucagon‐like peptide 1 (GLP1) or drugs like Victoza® (liraglutide), GLP1R undergoes internalization. Retaining GLP1R at the cell membrane contributes to a greater long‐term insulin release [10], and studying receptor trafficking dynamics and internalization rates can provide knowledge about its signaling properties and therapeutic potential [13]. For such targets mentioned above, the optical separation of protein pools is key to understanding protein trafficking and signaling dynamics as well as their functional lifecycles.

## Tags and labels

The most often encountered labeling tools for confocal, widefield, and super‐resolution microscopy consist of two components: a genetically encoded self‐labeling protein tag fused to the protein of interest (POI) and a bio‐orthogonal synthetic ligand conjugated to a (high‐performance) fluorophore. Fluorophore selection is typically within the visible spectrum, ranging from ultraviolet to far‐red light (laser lines e.g., 405 nm, 488 nm, 561 nm, and 638 nm), and need to avoid spectral overlap. Short‐wavelength fluorophores technically offer higher resolution but imaging may suffer from higher autofluorescence in tissue. Far‐red fluorophores provide deeper tissue penetration and reduced phototoxicity, however, usually display lower fluorescence quantum yields. The advantage of chemical‐genetic hybrids to conventional fluorescent proteins (e.g., GFP) is the greater photochemical flexibility and stability, with the downside that an exogenous fluorescent substance is required for visualization. Over the years, many different protein tagging systems have emerged, each offering distinct advantages for compartmental labeling. We focus on three common systems that have been used in several different experiments (Fig. 1A). The HaloTag Protein (HTP) is a 33 kDa modified haloalkane dehalogenase that forms a covalent ester bond with synthetic chloroalkane (CA)‐containing HaloTag ligands (HTL) [24]. It displays fast, diffusion‐controlled kinetics and exceptional stability of its ligands and has been (among others) implemented *in vivo* for the measurement of autophagic flux [25]. The SNAP‐tag is a 20 kDa mutant of the human DNA repair protein *O* ^6^‐alkylguanine‐DNA alkyltransferase which reacts rapidly and irreversibly with benzylguanine (BG) derivatives [26, 27], and has also been used *in vivo* for instance for cell ablation [28] or *ex vivo* for receptor trafficking [22]. The irreversible nature of the SNAP‐Tag and HTP labeling systems can, however, become a limitation in microscopy since high laser powers lead to photobleaching of the covalently attached fluorophore. Still, both tagging systems offer great potential for expression in mouse models using CRISPR‐based knock‐in approaches, enabling endogenous protein visualization while simultaneously preventing overexpression of artifacts [22, 29], which may comprise folding, trafficking kinetics, and signaling bias [30, 31]. Other tags are available that offer more layers of multiplexing, for instance the *O* ^2^‐benzylcytosine reactive CLIP‐Tag [32], eDHFR [33] that binds to trimethoprim in a non‐covalent but high affinity fashion (similar to recently reported FLEXTAG [34]), as well as short peptide tags like ybbR [35], ALFA‐ [36], HA‐ [37] and Spy‐Tag [38] that are more examples that represent the large repertoire in this field. Of note, exchangeable protein tags provide an option which allows for continuous replacement of the fluorescent dye during imaging, thereby circumventing photobleaching. Highlighting one system in this regard, the 14 kDa fluorescence‐activating and absorption‐shifting tag (FAST) functions by reversibly binding a non‐fluorescent fluorogen [39, 40]. The binding results in an increase in fluorescence and a red shift in absorption, allowing for high‐contrast imaging of FAST‐tagged proteins. In addition, reversibility introduces a unique advantage: the ability to “switch off” fluorescence by washing away the ligand, allows for dynamic labeling. Last but not least to mention, the BenzoTag [41], the OrthoTag [42] and the F_2_X system [43] were developed from HTP, respectively, while CLUSTER [44] is derived from a SNAP‐tag/split intein fusion and designed to limit tagging size.

**Fig. 1:**
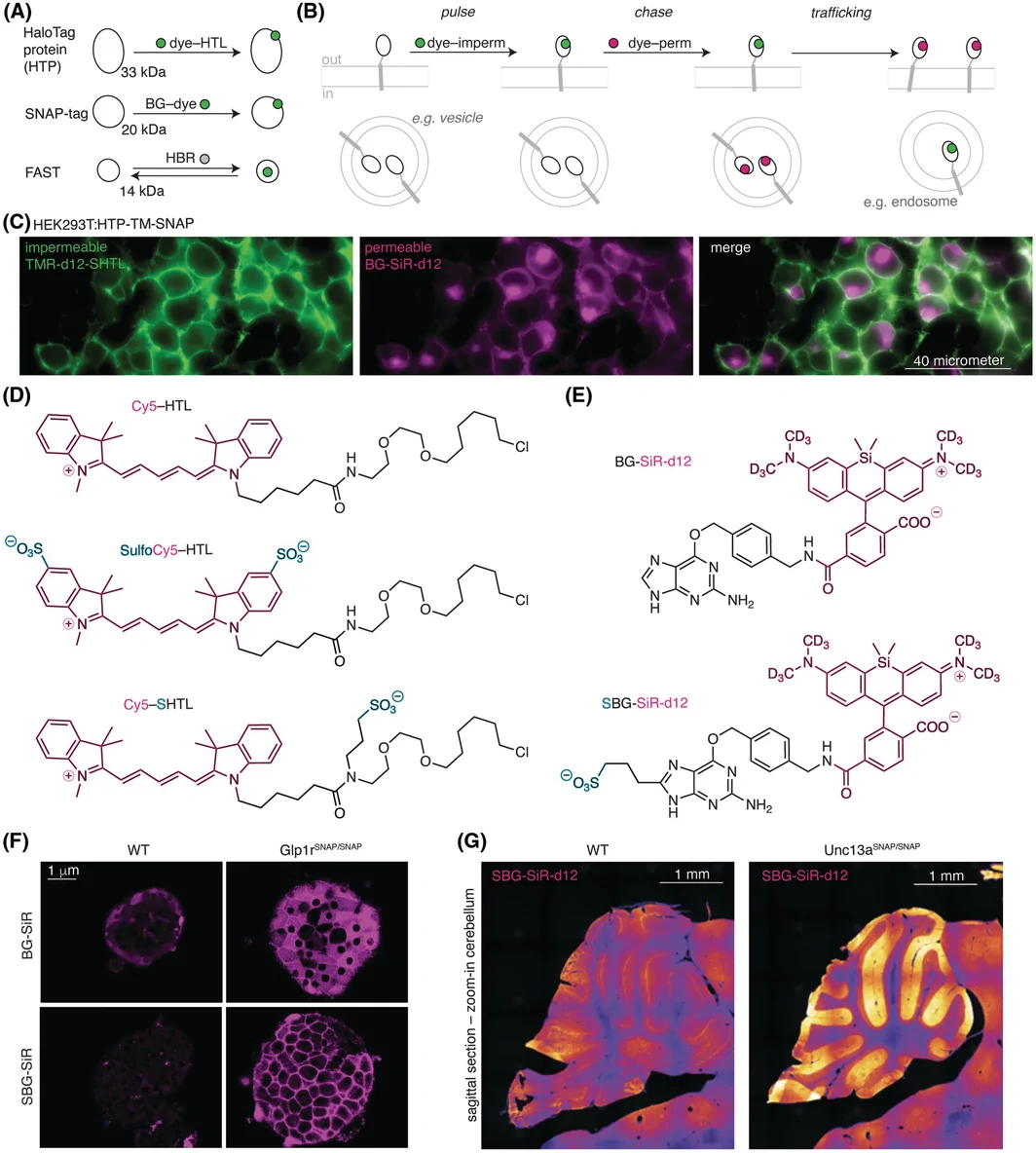
Strategies and applications for protein tagging and labeling. (A) Two self‐labeling proteins HaloTag protein (HTP, 33 kDa) and SNAP‐tag (20 kDa) covalently bind to HaloTag ligand (HTL) or benzylguanine (BG) conjugated to fluorophores, respectively. Fluorescence‐activating and absorption‐shifting tag (FAST, 14 kDa) binds an external fluorogen (e.g., hydroxybenzylidene rhodanine (HBR)), which becomes emissive only in a complex with the protein. (B) Schematic of pulse‐chase labeling using a self‐labeling protein tag and an impermeable and a permeable ligand, enabling multiplexing of different protein pools. (C) Microscopic image of HEK293T cells demonstrating the optical separation of internal and cell surface protein of interest (POI) populations (adapted from [21], under the terms of a Creative Commons CC BY 4.0 license). (D) Chemical structures of cell permeable Cy5‐HTL, dye‐sulfonated impermeable SulfoCy5‐HTL and ligand‐sulfonated impermeable Cy5‐SHTL. (E) Chemical structures of cell permeable BG‐SiR‐d12 and ligand‐sulfonated impermeable SBG‐SiR‐d12. (F) Confocal images of WT and *Glp1r* ^SNAP/SNAP^ knock‐in islet of Langerhans using BG‐SiR and SBG‐SiR (adapted from [22], under the terms of a Creative Commons CC BY 4.0 license). (G) Confocal images of WT and *Unc13a* ^SNAP/SNAP^ knock‐in sagittal slices of the cerebellum using BG‐SiR‐d12 and SBG‐SiR‐d12 (adapted from [23], under the terms of a Creative Commons CC BY 4.0 license).

## Embracing impermeability

Once a transmembrane POI is fused to the tag of choice and resides in both extra‐ and intracellular voids, the next step is to choose a fluorophore. Many high‐performing available organic dyes are membrane permeable to enter the cell's intracellular compartments. Unspecific background staining as well as accumulation of membrane permeable dyes in the cytoplasm or other compartments has been addressed by several research groups through fluorogenicity, prominent on rhodamine dyes like silicon rhodamine (SiR) [45], the JaneliaFluor [46, 47] or MaP [48, 49] series. Another approach was shown by the Urano laboratory by developing Oregon Green (OG) galactose derivatives based on fluorescein, for example a small fluorogenic molecule γ‐glutamyl hydroxymethyl rhodamine green (gGlu‐HMRG), that becomes fluorescent after enzymatic processing with γ‐glutamyltranspeptidase (GGT) [50]. However, this results in the labeling of all (surface‐exposed, inner biosynthetic, endocytic and recycling) membrane protein pools. In many functional studies regarding receptors, however, only the cell surface proteins are the target of interest to study their pharmacology, signaling, internalization rates and turnover. As such, researchers rely on using impermeable dyes (e.g., Alexa Fluor488, 568, or 647) to stain the cell surface exclusively (pulse), which in addition, can be followed with a permeable fluorophore (chase) to separate it from the intracellular pool (Fig. 1B) [17]. We have reported two protein fusions for straightforward benchmarking pulse‐chase experiments, comprising of HTP‐TM‐SNAP and SNAP‐TM‐HTP (TM = transmembrane) that may be expressed and display one tag on the outside, while the other remains in the cell (Fig. 1C) [51]. However, in times of super‐resolution techniques such as stimulated emission depletion (STED) and stochastic optical reconstruction microscopy (STORM), the choice of dyes is limited to the sophisticated techniques that are applied. Or in other words, a dye that outperforms in STED (e.g., SiR‐d12) is not adequately blinking for STORM [52] and a blinking dye for STORM (e.g., Cy5) might rapidly bleach in STED, limiting the available toolbox. As such, impermeability has to be applied according to the envisioned optical experiment. Jonker et al. used high‐performing rhodamine‐based JF_635_‐HTL and added two carboxylic acid moieties, thereby creating a cell impermeable JF_635_i‐HTL (i = impermeable) that allowed reliable performance in endocytic recycling assays and experiments requiring long‐term incubation (Table 1) [59]. Another chemical approach can be used on permeable HTL fused to cyanine5 (Cy5‐HTL, Fig. 1D): by the addition of a negatively charged sulfonate that repels the ligand from the cell surface, a high hydration shell is created around the molecule, making the energetic barrier for translocation through the hydrophobic lipid bilayer virtually impossible under physiological conditions. Sulfonates may be installed on the dye itself, giving SulfoCy5‐HTL [61], or may be added to the ligand, as we recently reported [21], giving the hypothetical Cy5‐SHTL (pronounced “shuttle” for SulfoHTL). Similarly for the SNAP‐tag, the high‐performing ligand‐fluorophore pair BG‐SiR‐d12 [62] may be rendered impermeable by fusing it to a sulfonated BG to obtain SBG‐SiR‐d12 (Table 1) [23, 53]. Elegantly, this sulfonate is part of the leaving group upon reaction with SNAP, thereby delivering the identical fluorophore as BG‐SiR‐d12, with the advantage that photophysics remain unchanged (Fig. 1E). When using dyes based on sulfonate‐mediated membrane exclusion, prolonged incubation times should be avoided as kinetics gradually drive intracellular accumulation over time, confounding quantification of truly endocytosed proteins. Measures can be taken to minimize this effect, such as short labeling times, treatment with endocytosis inhibitors (e.g., dynasore [63], bafilomycin A1 [64], Pitstop or Pitstop2 [65, 66]), and/or labeling at 4 °C [67]. Furthermore, a quantitative comparison of intracellular signal immediately after labeling (*t* = 0) compared to extended incubation times can provide a useful assessment of time‐dependent probe internalization [68]. SBG‐TMR (TMR = tetramethylrhodamine) and BG‐SiR were used in a pulse‐chase experiment to label extra‐ and intracellular pools in CHO‐K1 cells stably expressing SNAP‐GLP1R to follow receptor movement after stimulation with Exendin‐4 [53]. The SBG‐variants of several dye conjugates proved vital in a study to label the extracellular pool of an endogenous CRISPR/Cas9 SNAP knock‐in on the GLP1R ectodomain in live islets of Langerhans, an endocrine miniorgan within the pancreas (Fig. 1F), while the permeable BG‐SiR was predominantly staining intracellular pools [22]. It was confirmed that GLP1R is a specific beta cell marker for the insulin secreting cells, and using SBG‐TMR, it allowed to track receptor internalization with a set of peptidic agonists, such as Exendin‐4, semaglutide and tirzepatide, showing that GLP1R dynamics are heterogenous [22]. Using a BG‐SulfoCy5 ligand, direct STORM (dSTORM) was used to assess receptor nanodomains within primary tissue and cells [22]. The same receptor was labeled in a spatiotemporal manner by incorporating an unnatural amino acid photocage ONBY that was released under blue light, enabling no‐wash labeling at low concentrations of SNAP ligand BG‐Sulfo549 [69]. Notably, due to its higher solubility, SBG‐SiR‐d12 outperformed BG‐SiR‐d12 in a SNAP‐*Unc13a* knock‐in mouse in fixed and permeabilized brains, giving less background and stronger signals than its more sticky BG‐SiR‐d12 stablemate (Fig. 1G) [23]. Lastly, impermeable fluorogens have also been reported for the FAST by sulfonating the respective ligand hydroxybenzylidene rhodanine (HBR) (Table 1) [60]. These were used in membrane trafficking studies of the dopamine receptor D2 (D2R) fused to FAST and analyzed by flow cytometry in the presence or absence of the antipsychotic spiperone, a D2R antagonist, to interrogate trafficking defects of a D2R mutant [60].

**Table 1:** Summary of tagging systems.

Tag	Target	Ligand class	Cell permeable	Cleavable	References
SNAP‐Tag	Total protein	BG‐dyes	Yes	No	[46, 48]
	Extracellular pools	Sulfonated BG‐dyes	No	No	[51, 53, 54]
		Sulfo‐dye‐SS‐BG	No	Yes	[55, 56, 57, 58]
HTP	Total protein	HTL‐dyes	Yes	No	[46, 48]
	Extracellular pools	Sulfo‐dye‐SS‐HTL	No	Yes	[57, 58]
		SHTL‐dyes	No	No	[21]
		JFi‐HTL	No	No	[59]
FAST	Total protein	HBR	Yes	No	[39]
	Extracellular pools	Sulfonated HBR	No	No	[60]

*Note:* Protein tags and their respective dye‐conjugated ligand to resolve intra‐ and extracellular protein pools.

## Applications of sulfonated HaloTag ligands (SHTLs) in super‐resolution imaging and sparse labeling of mGluR2

The SHTL approach was successfully used to obtain SiR‐d12‐SHTL and, on a next generation HTL.2 [70], by double sulfonation to yield ATTO 647N‐S_2_HTL.2 (Fig. 2A) [21]. The former was used to stain and image the extracellular residing pool of HTP‐metabotropic glutamate receptor 2 (HTP‐mGluR2, Fig. 2B), a neuromodulatory GPCR, in lentivirally transduced hippocampal neurons. The pool on synapses was confirmed to be predominantly on the presynaptic site, by counterstaining with bassoon (presynapse) and SHANK2 (postsynapse) antibodies using confocal imaging (Fig. 2C). As SiR‐d12 is an outstanding dye for STED nanoscopy due to its brightness and photostability, super‐resolution images of HTP‐mGluR2 positive processes allowed resolving fine structures next to synapses (Fig. 2D) with a resolution of 134 nm (Fig. 2E). Introducing sulfonates on ATTO 647 N‐HTL.2, making ATTO 647 N‐S_2_HTL.2 counteracted the dye's intrinsic stickiness. As such, when compared in labeling and imaging of HTP‐SNAP‐mGluR2 transfected HEK293T cells, the non‐sulfonated version led to non‐specific binding, while the double sulfonation gave clear cell surface signals. The second generation HTL.2, which was initially designed to be able to titrate down concentrations for HTP labeling [70], allowed in sparsely transfected systems (12.5% amount of DNA than normally used) and with brief labeling times (10 min before fixation) HTP‐mGluR2 signals detectable with 1 nm labeling concentrations (Fig. 2F). Together, these two examples demonstrate the usefulness of sulfonation across a diverse set of systems.

**Fig. 2:**
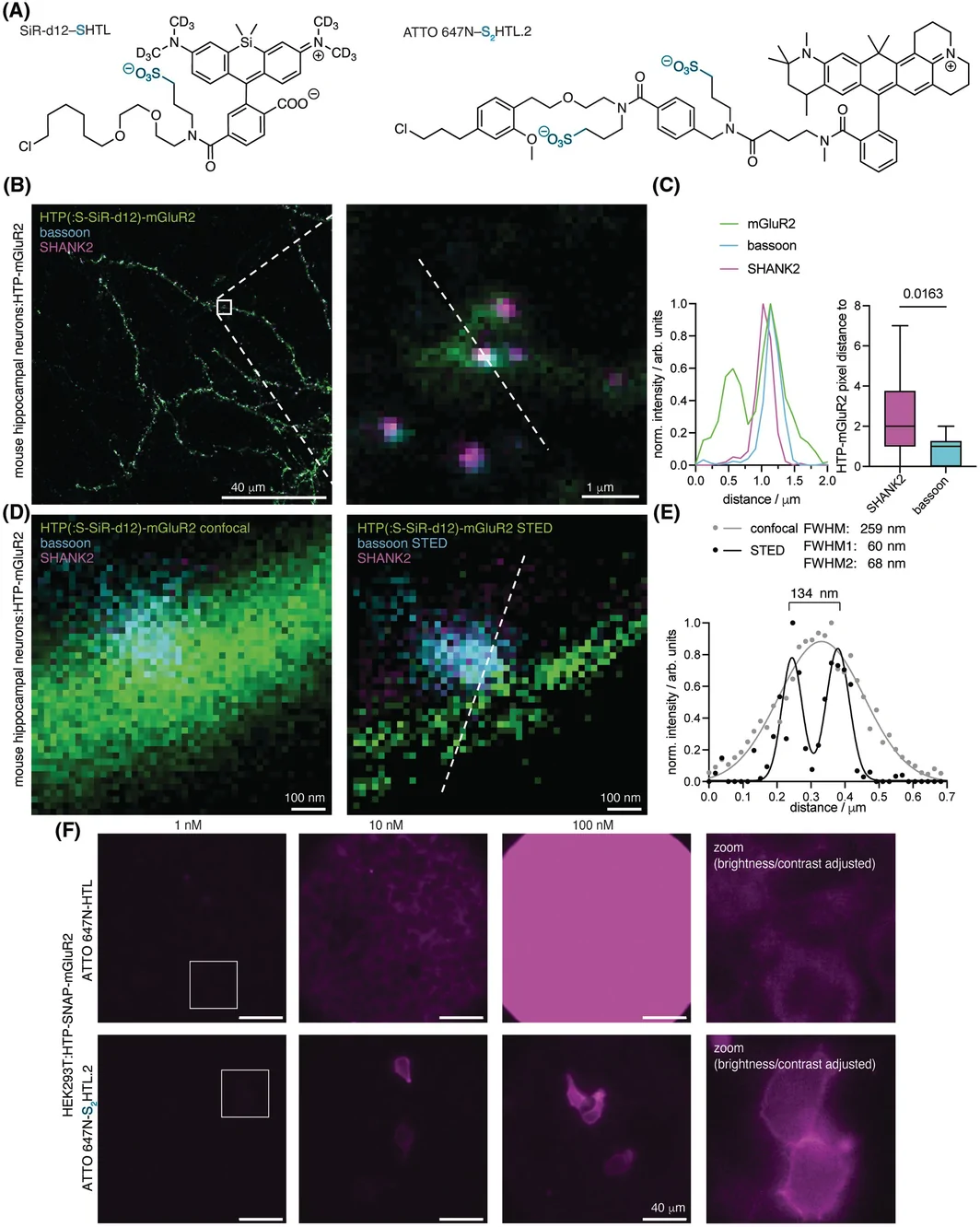
Impermeable sulfonated HaloTag ligands (SHTLs). (A) Chemical structures of ligand‐sulfonated SiR‐d12‐SHTL dye and double ligand‐sulfonated ATTO 647N‐S_2_HTL.2. (B) Confocal image (and zoom in with line scan) of hippocampal neurons transduced with HTP‐metabotropic glutamate receptor 2 (HTP‐mGluR2) and labeled with SiR‐d12‐SHTL, bassoon (presynapse) and SHANK2 (postsynapse). (C) Line scan from (B) reveals main extracellular mGluR2 is presynaptic. (D) Confocal and stimulated emission depletion (STED) images of neurons from (B) across HTP‐mGluR2 positive processes, including line scan. (E) Line scan from (D) shows sub‐diffraction limit resolution. (F) HTP‐SNAP‐mGluR2 transfected HEK293T cells labeled with different concentrations of ATTO 647N‐HTL and ATTO 647N‐S_2_HTL.2. ATTO 647N‐S_2_HTL.2 reveals the sulfonated version to be non‐sticky and specific. B‐F adapted from [21], under the terms of a Creative Commons CC BY 4.0 license.

## Removal of cell surface labels

Another emerged method to study cell surface receptor trafficking events involves the use of a post‐labeling cleavable substrate for the self‐labeling protein tags (Fig. 3A) [55, 56, 57]. The substrate is impermeable, exclusively labeling receptors on the cell surface. As the receptor undergoes agonist‐evoked endocytosis and intracellular trafficking, two protein pools are separated to non‐internalized receptors and receptors in the endosomal/lysosomal system, the former of which can be confounding when studying endocytosis and recycling dynamics of internalized protein pools. Accordingly, substrates are designed to contain a disulfide bridge (SS) as a linker between the dye and the impermeable substrate, like SulfoCy5‐SS‐HTL (or former CA‐SS‐SulfoCy5) (Fig. 3A) [57]. This disulfide bond can be cleaved using an impermeable reducing agent MESNA (sodium 2‐mercaptoethane sulfonate). Short incubation of 15 min results in the rapid and irreversible stripping of dyes from remaining membrane‐residing receptors, only leaving the internal receptor pools labeled (Fig. 3A). Thus, the recycling dynamics of endocytic pools as well as quantitative measurements of internalization can be studied in live and/or fixed cells using microscopy [57]. However, certain caveats need to be considered that may confound the interpretation of trafficking or surface‐labeling experiments. Although MESNA is widely regarded as membrane‐impermeable, its exclusion from the intracellular space is not absolute: prolonged incubation times, high concentrations, or compromised membrane integrity may permit intracellular access [71]. And, more generally, reducing conditions may cleave native disulfide bonds, and thus, controls such as no‐MESNA and non‐cleavable analogues as well as viability assays (e.g., WST‐1) should always be included in the experimental design.

**Fig. 3:**
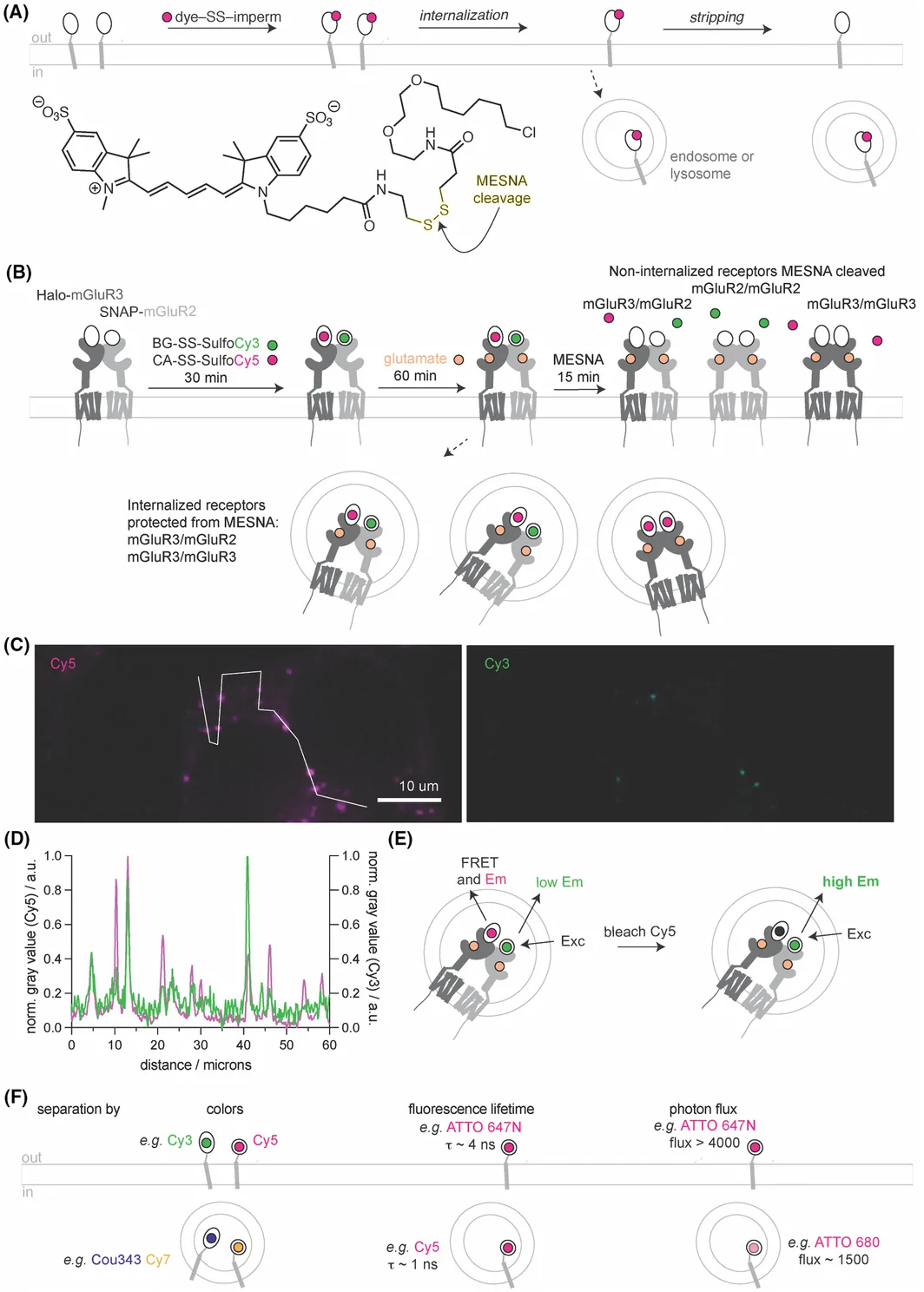
Cleavable dye‐ligand conjugates allow stripping of extracellular labeled protein pools and emerging separation techniques. (A) Schematic representation of a stripping experiment on HTP‐protein of interest (POI) transfected cells binding to a disulfide‐containing dye‐SS‐impermeable ligand, followed by partly internalization and stripping of the cell surface remaining pool with sodium 2‐mercaptoethane sulfonate (MESNA). Chemical structure shows SulfoCy5‐SS‐HTL including the molecular cleaving site. (B) Schematic of SNAP‐metabotropic glutamate receptor 2 (SNAP‐mGluR2) and HTP‐mGluR3 co‐expression and labeling with their respective probes, before agonist glutamate leads to internalization of mGluR3 containing receptor dimers, and addition of MESNA cleaves fluorophores of remaining extracellular receptors. (C) Images from experiment described in (B) including line scan. (D) Line scan from (C) shows Cy3/Cy5 co‐localization in intracellular compartments. (E) Schematic that heterodimers can be used for Förster resonance energy transfer (FRET) measurements. (F) Classical separation of pools is achieved spectrally (left), but can also be performed with one colour.

## Further possibilities and future developments

While so far, we focused on the selective analysis of different pools of a single protein, this concept can be extended to multiple target proteins simultaneously through multiplexing, using different self‐labeling proteins. For example, one POI is expressed with a SNAP‐tag, while a second POI is expressed with a HTP. By using the respective substrates with varying, spectrally distinct fluorophores, both proteins can be visualized in parallel (Fig. 3B). This approach enables the simultaneous analysis of co‐internalization and co‐localization, and recent studies have utilized this approach to investigate protein interactions. Roßmann et al. used multiplexed labeling strategies to co‐localize mGluR homo‐ and heterodimers [57]. As per, SNAP‐mGluR2 and HTP‐mGluR3 were co‐expressed in HEK293T cells and labeled with cleavable fluorescent probes BG‐SS‐SulfoCy3 and HTL‐SS‐SulfoCy5. Subsequent treatment with glutamate led to the activation of the mGluR2 and mGluR3 and facilitated internalization of only mGluR3‐containing dimers. Cleavage of the remaining cell surface fluorophores with cell impermeable MESNA enabled the visualization of the trafficked receptors in intracellular compartments (Fig. 3B,C). Providing line scans allowed to assess receptor co‐localization (Fig. 3D), and potential receptor crosstalk can be probed by bleaching the acceptor molecule, abolishing Förster resonance energy transfer (FRET) (Fig. 3E), which only occurs when the donor and the acceptor are within the range of 3–10 nm. This has been demonstrated on mGluR subunits to validate receptor heteromerization [57, 72]. To validate the co‐localization, acceptor photobleaching is often used for intentionally bleaching the acceptor (e.g., Cy5) with a high‐power laser, which in turn increases the emission of the donor (e.g., Cy3).

## Different ways of separation

Confocal, widefield, and super‐resolution microscopy offer a powerful multiplexing tool, but limit the number of protein targets that can be visualized simultaneously down to the spectral channels available (usually four). To expand the possibilities of multiplexing in microscopy, fluorescence lifetime imaging microscopy (FLIM) is another technique which distinguishes between chromophores based on their mean fluorescence lifetime (Fig. 3F, left). This enables the use of several fluorophores (e.g., Cy5 and ATTO 647N) in one spectral channel, which allows for the separation of multiple different cellular targets: Frei et al. demonstrated the spectrally resolved detection of six targets in living cells simultaneously by the use of different HTP variants and only two spectral channels [73]. But temporal resolution is limited in live‐cell imaging due to high laser power and long acquisition times, which lead to phototoxicity. FLIM, as such, opens up the possibility to visually separate intra‐ and extracellular protein pools. For this, each POI needs to be expressed with a different self‐labeling tag (e.g., SNAP‐tag and HTP) and labeled with a respective permeable and impermeable substrate in the same spectral channel by pulse‐chasing (Fig. 3F, middle). This would enable the pairwise visualization of protein pools in parallel, thereby doubling (or even tripling) the number of targets compared with conventional microscopy. However, the local environment of intra‐ and extracellular targets needs to be taken into consideration carefully when selecting the chromophores, as fluorescence lifetime is sensitive to pH, ion concentration, and viscosity (for a timely review, see ref [74]), which could confound assignment to a protein pool rather than the respective tag. Since fluorescence lifetime is measured in FLIM, the demands on the photon budget are especially challenging when using weak dyes, since high quantum yield and photostability are required for accurate measurements [75].

Lastly, spectral demixing in STORM (SD‐STORM) and DNA‐points accumulation for imaging in nanoscale topography (DNA‐PAINT) offer an additional tool for multiplexing—techniques in which multiple dyes within the same emission spectrum (e.g., ATTO 647N, ATTO 680) are excited by a single laser but can be distinguished based on their intrinsic photon flux (intensities) (Fig. 3F, right) [76]. SD‐STORM was employed by Le et al. to label three different cellular targets simultaneously (i.e., microtubules, clathrin, and vimentin), giving a 3‐colour super‐resolution image by only using a single channel [77]. Similar to FLIM, SD‐STORM also demands a high photon budget, which makes the selection of suitable dyes challenging [78]. While offering multicolor imaging with high spatial resolution, major drawbacks of SD‐STORM are long acquisition times which promote photobleaching and sample drift [79]. The latter of which requires extremely accurate channel alignment and calibration tools and software has been developed [80]. In comparison, DNA‐PAINT is easier to implement and technical requirements are lower [81]. However, limitations apply as DNA‐PAINT is exclusively applicable to fixed samples and if the use of antibody conjugation is required, this results in a noticeable linker error [82]. Ultimately, this concept was transferred to self‐labeling proteins: Kompa et al. developed exchangeable ligands for the HaloTag (xHTLs) which unlocked HaloTag compatibility for “PAINTing” [83].

## Summary

Many powerful tools exist in modern microscopy to visually separate protein pools for the study of transmembrane protein dynamics. While protein tags and the use of permeable and impermeable ligands in pulse‐chase experiments enable the precise labeling of such, super‐resolution microscopy, FLIM, and spectral/brightness demixing expand the capacity for parallelization. Together, this review highlights few of the current developments, depicts the broad range of possibilities in the field, and is looking forward to future methods to come.

## Author contributions

KF: visualization, writing—original draft preparation. JB: funding acquisition, supervision, visualization, writing—original draft preparation.
